# Shaping Pathways to Child Health: A Systematic Review of Street-Scale Interventions in City Streets

**DOI:** 10.3390/ijerph19095227

**Published:** 2022-04-25

**Authors:** Adriana Ortegon-Sanchez, Laura Vaughan, Nicola Christie, Rosemary R. C. McEachan

**Affiliations:** 1 Centre for Transport Studies, Department of Civil, Environmental and Geomatic Engineering, UCL, London WC1E 6BT, UK; nicola.christie@ucl.ac.uk; 2 Space Syntax Laboratory, The Bartlett School of Architecture, UCL, London WC1E 6BT, UK; l.vaughan@ucl.ac.uk; 3 Bradford Institute for Health Research, Bradford Teaching Hospitals NHS Foundation Trust, Bradford BD9 6RJ, UK

**Keywords:** built environment, streets, interventions, children, deprivation, health, playstreets, play streets

## Abstract

Street-level built environment factors, for example, walking infrastructure, building density, availability of public transport, and proliferation of fast-food outlets can impact on health by influencing our ability to engage in healthy behaviour. Unhealthy environments are often clustered in deprived areas, thus interventions to improve the built environments may improve health and reduce inequalities. The aim of this review was to identify whether street-level built environment interventions can improve children’s health in high income countries. A secondary aim was to describe key built environment elements targeted in interventions and research gaps. A systematic review of published literature was conducted by a multi-disciplinary team. Ten intervention papers were included. Physical activity or play was the only health outcome assessed. Most interventions described temporary changes including closure of streets to traffic (N = 6), which were mainly located in deprived neighbourhoods, or the addition of technology to ‘gamify’ active travel to school (N = 2). Two studies reported permanent changes to street design. There was limited evidence that closing streets to traffic was associated with increases in activity or play and inconclusive evidence with changes to street design and using technology to gamify active travel. Our ability to draw conclusions was hampered by inadequate study designs. Description of interventions was poor. Rigorous evaluation of built environment interventions remains challenging. We recommend a multi-disciplinary approach to evaluation, explicit reporting of built environment indicators targeted in interventions and offer solutions to others working in this area.

## 1. Introduction

Non communicable diseases (NCDs) such as cardiovascular disease (for example, heart attacks and stroke), cancer, respiratory disease and diabetes kill an estimated 41 million people globally each year, and yet are largely preventable [1]. A healthy lifestyle (for example, having a healthy diet, exercising, avoiding smoking or alcohol use), or lack thereof, is a key contributor to our risk of developing NCD [1]. Childhood is a critical time period for improving health, as evidence shows that early life exposure to key stressors can affect disease risk in later life [2]. Healthy behaviour patterns that are established in childhood [3] and adolescence track into adulthood [4], and thus encouraging a healthy lifestyle can have benefits across the life-course.

In high income countries, the burden of NCDs fall more heavily on communities living in deprived areas [5], due to a conglomeration of interrelated risks. Unhealthy lifestyle patterns are more prevalent in deprived communities which means these groups are more likely to develop physical health conditions such as raised blood pressure and obesity that are precursors to NCDs [6]. Families in deprived areas are likely to experience greater stresses that are in turn related to unhealthy behaviour [6]. Finally, communities in deprived areas are likely to be living in areas with a range of environmental risk factors such as pollution [6], lack of access to high quality green space [7,8], and high density/poor quality built environments [9], which can both directly, and indirectly via behaviour, influence the progression of disease [6].

Recognition of the importance of the built environment in determining health outcomes is nothing new. Indeed, poverty, impoverished environments, and poor health was observed by many of the earliest medical researchers, with social reformers such as Octavia Hill recognising the importance of preserving the common lands and parks to provide lungs for city dwellers [10]. These environmental injustices serve to heighten health inequalities and, as they are spatially persistent, are hard to address. It is very difficult to change patterns of spatial injustice in the city, such as the location of polluting factories, unsafe roads, or lack of access to parks. For example, in a study of mortality in relation to poverty in childhood, it was found that “the fundamental relation between spatial patterns of social deprivation and spatial patterns of mortality is so robust that a century of change in inner London has failed to disrupt it” [11].

The difficulties in changing environments means that historically, efforts to improve lifestyle behaviours have targeted individual behaviour change [12], which is problematic as it assumes equal agency between different population groups. For families living in deprived areas where there are structural barriers, such as constraints on walking, fast food swamps, high levels of traffic, lack of green space, or fears about safety, it can be more difficult to lead a healthy lifestyle. The risk then is that policies focusing on individual behaviour change only serve to heighten health inequalities [13], with more affluent groups being better able to take advantage of preventive activities, thus improving health, whilst less affluent groups are left behind. Interventions which aim to improve environmental determinants of health have the potential to reduce inequalities, if efforts are targeted in areas of most need.

Our own recent meta-narrative review of 108 studies [14] exploring associations between built environment indicators and health outcomes found ten built environment categories implicated in children’s health. These included residential density, street connectivity, land use diversity, walkability, pedestrian infrastructure, physical activity facilities, availability of open space, safety from traffic and crime, traffic levels and social support for undertaking activities such as active travel. Health outcomes typically measured included physical activity or active travel, and obesity. However, we found a wide variation in the ways in which built environment indicators were conceptualised and measured across different disciplines which hampered interpretation of the literature. We posit that it is important to understand the built environment at ‘street scale’ to appropriately capture the characteristics of the built environment at the scale at which they are experienced by users on the ground.

Previous reviews of intervention studies have suggested a potential role for environmental factors in influencing health behaviours, predominantly physical activity. In an umbrella review covering a variety of place-based interventions, McGowan et al. identified infrastructure changes related to housing, provision of active travel infrastructure, public transport and amenities, and green space to be potentially linked to health outcomes, in particular, physical activity [15]. They raised a note of caution in that some of these ‘place-based’ interventions required active change from local residents in order to improve health. For example, improvements to cycle paths are only beneficial if residents actively use the new facilities [15], and improvements to green spaces will result in increased use only if communities feel safe using these spaces [16].

Three reviews focusing specifically on urban green space interventions found some evidence that multi-component interventions combining infrastructure changes with other approaches designed to encourage use of green space were effective in increasing physical activity amongst children and adults [17,18,19], and that involving communities in redesigning spaces was associated with increases in park use [19], but that there was too little evidence to draw conclusions about whether these interventions had a measurable impact on health inequalities [18].

There has been limited research focused on built environment interventions targeted at young people. In their review, Audrey and Batista-Ferrer [20] found some evidence to support interventions to reduce road traffic injuries (e.g., 20 mph zones or walking infrastructure improvement), and active travel interventions (including changes to the built environment) as important for children. Similar to Hunter et al. [18], they reported that few studies explicitly examined impact on health inequalities. All of the reviews discussed above highlighted a number of methodological issues with the types of evaluations presented likely to introduce substantial sources of bias, for example, non-randomised designs, lack of control groups, quality of outcome measures, literature which hinders our ability to make conclusions about their utility. Further, Roberts et al. [19] commented on the inadequate description of intervention content, potentially hindering replicability.

The aim of this study was to review the evidence of built environment interventions on children’s health outcomes. We chose to base our review within high and upper-middle income countries as evidence shows that deprived communities experience greater health inequalities in these settings, and thus there is greater potential for impact for effective built environment interventions.

Importantly, our intention was to capture studies of how the urban built environment interacts with children’s lives, using keywords that relate to streets, in order to capture studies that considered the built environment as a measurable, human-scale environment. We also aimed to describe key intervention features and quality of studies and provide recommendations for researchers working in this area.

## 2. Materials and Methods

This systematic review of published literature was completed by a multidisciplinary team. Given the methodological considerations raised in previous reviews, we kept our inclusion broad to capture a range of study designs. A PICO summary was created to guide the review process as follows:

Population of interest: Children aged under 18 living in high and upper-middle income countries

Intervention: Any intervention which involved physical changes to street-level built environment. We defined the built environment as the complex space created by the interaction of various physical structures that support human activity such as roads and streets, pavements, buildings, street furniture or open spaces, among others.

Control: Any study design was included—it was not necessary to have a control arm

Outcome: Child health outcomes (for example reported Body Mass Index—BMI) or activities (for example observed park use, parent-reported play or active travel to school).

### 2.1. Search Strategy

A structured keyword search was conducted in four relevant databases, Medline and Embase (Medical and Biomedical sciences), PsycInfo (Behavioural and Social sciences) and Scopus (Physical sciences, Health Sciences, Social Sciences and Life Sciences) on 8 November 2021. Based on their knowledge of the literature in their fields, the research team identified the keywords for the search within four concepts: (i) Streets, (ii) Built Environment (including urban open spaces or infrastructure, public spaces, or land use), (iii) Health Activities and Health Outcomes and (iv) Children. The full search strategies for all databases are presented in Supplementary Material S1.

### 2.2. Study Inclusion Criteria

Studies were eligible for full-text review if they included (i) objective or standardised subjective measures of streets and the built environment; (ii) objective or self-reported measures of children’s physical activity or health; (iii) considered a permanent or temporary change or intervention to the built environment; and were (iv) completed from 2010 onwards in upper-middle and high-income countries accordingly to the World Bank classification (http://data.worldbank.org/about/country-classifications, accessed on 8 November 2021). Studies were excluded if they reported (i) interventions which did not change the built environment but only considered changes to policies and/or programmes, (ii) interventions exclusively related to cycling and food environments, or (iii) were reviews, protocols or studies to validate tools or methods or were studies not published in English. Conference papers, books and grey literature were not eligible for inclusion but were inspected to identify relevant references.

### 2.3. Data Extraction

Two researchers (A.O.-S., R.R.C.M.) extracted the following main characteristics: authors, publication year, study area, study location, study design, sample size, sample age, health activities/health outcomes, methods of analysis and key findings. Another researcher (RM) extracted information on intervention characteristics using the TIDieR (template for intervention description and replication) checklist [21]. Missing information was reported.

### 2.4. Quality Assessment

Risk of bias and study quality was assessed using a tool adapted from similar reviews of environmental interventions [18,22]. Two reviewers (A.O.-S., R.R.C.M.) independently scored the included studies on eleven criteria. The two independent quality assessments resulted in initial agreement for seven out of ten studies. Disagreements were resolved by discussion. One criterion (attrition) was only scored where studies reported relevant study designs (for example, more than one study period). One point was awarded if the study met the criteria, thus studies could score between 10–11 points. In line with previous reviews, a score of >9 was considered high quality (see full details of the quality assessment criteria in Supplementary Material S2 Table S2.1). The assessment was completed to methodically appraise the risk of bias and uncertainty in the results presented by the reviewed studies. However, the scores were not used as an exclusion criterion as, based on previous reviews, it was anticipated that few studies would be categorised as high quality, considering the intrinsic difficulties associated with the evaluation of built environment interventions.

## 3. Results

### 3.1. Study Selection

**Figure 1:**
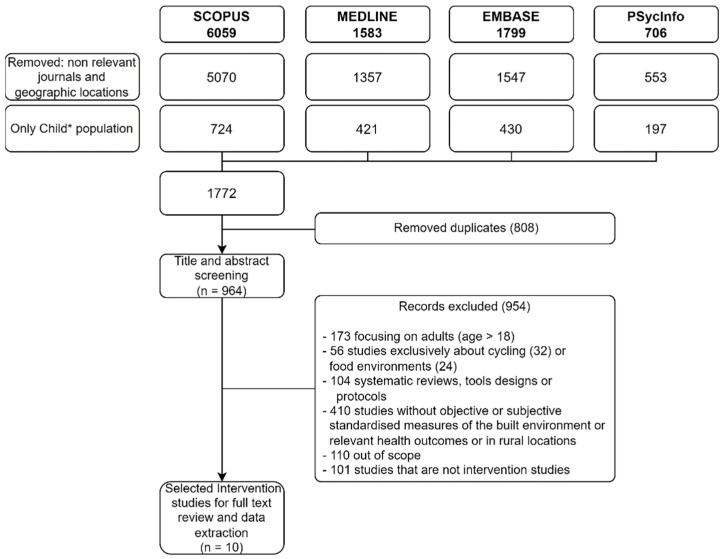
Flow diagram of study selection. (* indicates truncation at the end of the word Child to expand the search to include any ending of the root word child, such as child’s, children, childhood).

The search retrieved 941 studies, after removing duplicates, all of which were screened against the eligibility criteria in Title, Abstract and Keywords by two authors. Ten studies were identified as eligible for full-text review (Figure 1).

### 3.2. Study Characteristics

The ten studies found all involved an intervention to change the street-level built environment and an attempt to evaluate the impact on health outcomes amongst children. Table 1 provides details on the studies. Most were conducted in the USA [23,24,25,26] with the remainder in the UK [27,28], Belgium [29], Germany [30] and Chile [31]. One paper reported a multinational intervention conducted across the UK and Canada [32].

The interventions described fell into three main categories: (i) street closure interventions: play streets, involving the temporary closure of streets to motorised vehicles to facilitate outdoor play, physical activity or cycling [23,24,25,26,29,31]; (ii) street design interventions: design features of the built environment to promote health [27,30]; (iii) walk to school technology interventions: addition of temporary technology (sensors and swipecards) to incentivise walking to school [28,32]. Five studies explicitly noted that interventions were conducted in areas of high deprivation [23,25,26,30,31]. Four of these were street closure studies. Of these, three studies were reported to have been completed in low-income areas in the US in settings such as Columbus, Ohio [23], and in areas with higher-than-average rates of disease in Brownsville, Texas [25] and San Francisco [26]. One study was completed in low-income neighbourhoods in Santiago, Chile, in an area also characterized by drug dealing issues [31]. Of the two street design interventions, one was developed in a large housing estate in Leipzig, Germany, with above average unemployment rates, low education levels and below average income levels [30].

The age range of children included in the evaluation was specified in five studies, the most common distinctions were between pre-school, children, and teenagers. Adult presence was a consideration in all cases, with some instances taking account of adults as part of a broader analysis of how families respond to an intervention (e.g., [25]), with others focusing on adult supervision of younger children (e.g., [27]). Most recorded male/female numbers, but only five of the ten quantified sex differences. Ethnic differences were recorded and analysed only in one case [25]. The total number of recruited child and teenager participants reported in the papers ranged from 80 [28] to 3817 [32]. Since many of the interventions were temporary events, various studies reported observed participants over the duration of the event as sample sizes; these varied from 293 [31] to 2577 [25].

Primary outcomes assessed in studies centred on physical activity conceptualised as outdoor play or activity [23,24,25,26,27,30], moderate to vigorous physical activity [28,29], stepcount [31], and active travel/walking to and from school [32]. Key tools to measure physical outcomes included objective instruments such as accelerometers [28,29], pedometers [31] and swipe card technology [32], standardised observations tools such as the SOPARC [24,25,26,30], bespoke observation tools [27], or self-reported activity [23,28,31,32]. Some also used surveys or interviews to capture attitudes towards the intervention [23,26,32].

The level of detail in describing the built environment ranged from the use of geographical data to capture the amount of additional open spaces for play [26], to more descriptive accounts of available amenities and facilities, land-uses (mixed or residential), and type and conditions of housing [27,31]. The latter also described the permeability and connectivity of the sites, distinguishing between streets that were cul-de-sacs and those that were not; streets with designated play areas, and those that simply made it possible by eliminating cars. Biddulph (2012) also measured traffic speed across the sample and provided maps for each of the areas where the intervention was being implemented. The two travel-to-school interventions [28,32] did not systematically describe the built environment.

#### Study Design and Risk of Bias

Generally, risk of bias was high, with no studies reaching the threshold of 9/11 for ‘high quality studies’ identified in previous reviews. The predominant missing aspects in the studies were those related to randomisation, exposure and representativeness. No studies used randomisation to assign exposure, and no studies explored whether there was evidence of a concurrent intervention which may have influenced the results. The representativeness of the study populations included in the review were insufficiently described in all included studies.

The strongest papers (scoring > 7/8) reported quasi-experimental studies which included a pre-test/post-test design either with ([31] 8/11, [29] 7/11, [28] 8/11) or without a control group ([30] 7/10). One paper reported a pre–post test design without a control group ([32] 4/10), but scored lower on the quality assessment because the lack of control limited the comparability of baseline characteristics, high attrition (50%) and follow-up was completed immediately post-intervention. One study reported a controlled post-test design ([26] 5/11). Four studies reported post-test evaluations with no control group ([23] 2/10, [27] 4/10, [24] 3/10, [25] 5/10). Supplementary Material S2 (Table S2.2) contains details of risk of bias for the included studies.

### 3.3. Description of Interventions

Table 2 summarises the key features of the included interventions. A full description of the interventions using The Template for Intervention Description and Replication (TIDieR) can be found in Supplementary Material S3 (Table S3) [33].

The six street-closure interventions generally aimed to create safe opportunities for outdoor play for children and communities. These interventions targeted four separate built environment categories identified as important for children’s health [14]: increasing availability or proximity to public open and social spaces, increasing perceptions of safety from traffic and crime, reducing traffic and promoting social support, and other psychosocial factors.

These interventions were all temporary and ranged in frequency including regular events (e.g., twice a week for 12 weeks [31]; every 2 weeks for 2 months [23]; 4–6 times a year [25] to more ad-hoc events [24,26,29]. When implemented, the street closures were in place for a number of hours and tended to be held during the summer months. Closures were mainly within residential blocks, although one study reported city-wide closures of roads totalling 2–3 miles to link up key city parks. Alongside the street closures, a variety of additional activities were implemented. These were primarily based on community preferences and included, for example, organised sports activities for children, providing of entertainment, or provision of information regarding other services available within the area. Additional equipment was often provided, either for group entertainment (e.g., inflatables, such as bouncy castles) or for individuals (e.g., sports equipment). Some playstreet initiatives were directly led by communities who had to apply to be able to hold the event [29]. Other initiatives tailored the activities to community preferences via regular meetings with community representatives [26,31]. It was unclear how communities were engaged in three of the studies [23,24,25]. Some play streets were supported by commissioned local community members [31], community hosting organisations [24] or non-profit organisations [26]. Most reported using volunteers to help hold the events, for example by helping to enforce the street closure or by holding additional activities. Two studies reported police involvement in helping to patrol streets and enforce road closures. The costs of holding the events were rarely reported, with the exception of one study [31], who reported that overall costs for 26 playstreet sessions was US $2275. Pollack Porter et al. [24] mentioned seed grants of between US $4000–5000 paid to two delegate agencies who then selected hosting partners in their respective regions. From this budget, delegate agencies provided hosting partners with seed grants of up to US $1000 to cover staff stipends, food, and money for materials (e.g., jump ropes).

The two intervention studies which implemented permanent changes to the environment with specific design features did not include any additional community activities. In terms of built environment categories, these targeted pedestrian infrastructure or street environment design, and to the extent that they encouraged communities to use these spaces, they could also increase social support indirectly. The latter type of intervention varied in scope, with the most comprehensive reported by Biddulph [27], which described environment changes to reprioritise streets to make them safe places to play. The types of changes implemented here included having shared surfaces, with no clear priorities for cars or pedestrians, with various types of street furniture designed to encourage individuals to spend time in the environment. The changes were implemented by local developers and local community engagement was not reported. Igel et al. [30] described implementation of movement-enhancing footpaths to create attractive places for children to play. This included a permanent decoration for children’s street games which was intended to encourage play. This change was implemented by a community-based health project and was the result of participatory planning process with students from two local primary schools to help decide on the final design. No costs were reported for either intervention reported by the authors.

Finally, two studies [28,32] explored the implementation of a ‘beat the street’ intervention which was targeted at school children to encourage active travel to school. Reflecting on the 10 built environment categories identified previously [14], these interventions targeted only the social support and psychosocial factors category. The interventions included temporary changes to the local environment around schools by adding ‘beat box’ sensors to key locations on the route to and from school. Participants were given a swipecard and ask to touch the sensor with their card on the walk to school. This intervention was supported by other activities including competitions between schools based on points accrued and provision of incentives. These interventions were delivered in school and length ranged from four weeks [32] to nine weeks [28]. It was unclear whether schools were involved in the development of the intervention, although some engagement was reported, where schools developed their own in-house competitions and rewards [32]. The costs of implementing these interventions were not reported.

### 3.4. Impact of Interventions

**Figure 2:**
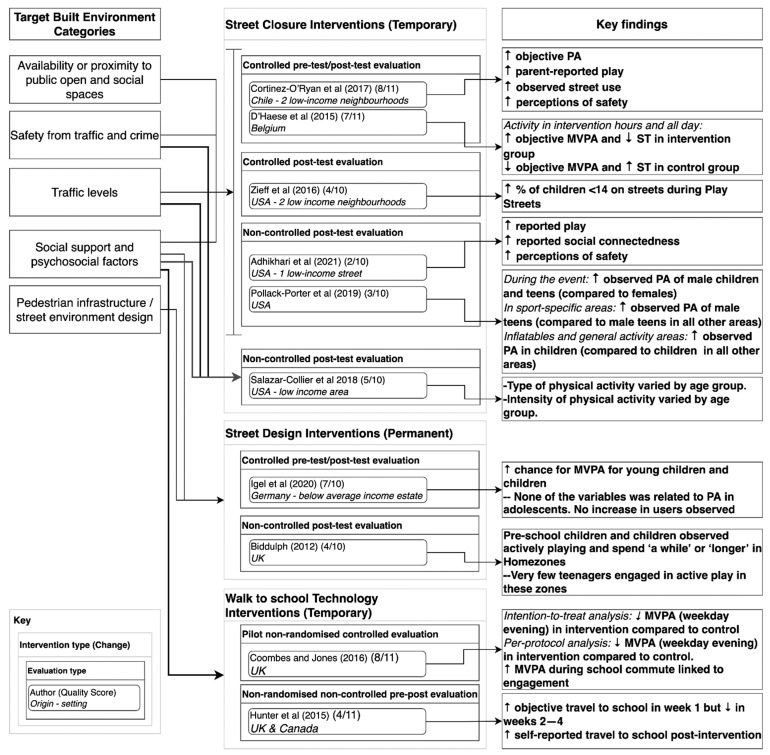
Targeted built environment categories, interventions and key findings.

Due to the diversity of study designs and measurements, we were not able to summarise findings quantitatively. Below we present a narrative review for each type of intervention, studies with a stronger design are discussed first. Full details can be found in Supplementary Material S4 (Table S4), and the results are summarised in Figure 2.

#### 3.4.1. Street Closure Interventions

Of the two controlled pre-test/post-test evaluations, Cortinez-O’Ryan et al. [31] found a significant increase in the number of steps children took during weekdays and during intervention hours as assessed via a pedometer, and a corresponding increase in parental self-reported daily and weekly outdoor play. D’Haese et al. [29] found a significant effect of play streets on sedentary behaviour assessed via accelerometry. In the intervention group, sedentary behaviour was lower (138 min/day) when play streets were being implemented than on a normal day than on the non-intervention week (146 min/day). In the control group, sedentary behaviour was higher during the intervention week (164 min/day) than the non-intervention week (156 min/day).

In their post-test controlled evaluation Zieff et al. [26] found that the percentage of children below 14 years of age on the streets increased from 5% to 38% during play streets compared with a comparison day. They also found that there were more instances of children engaging in vigorous physical activity outside as assessed via observations on playstreet days versus comparison days. They did not find much engagement amongst teenagers and they concluded that the focus of the intervention on families might have discouraged teenager involvement.

Two play street interventions reported only post-test, non-controlled evaluations, making it difficult to attribute any patterns in physical activity to the intervention. Adhikhari et al. [23] reported that of 69 caregivers surveyed at the event, 55% said their children played outside on 5–7 weeks, and 16% said they played out 1–2 days a week. They reported that 55% of caregivers said their children would be playing inside if not for the playstreet. Fifty-three percent reported that their children played more as a result of the playstreet. They also reported a range of ancillary benefits including children making new friends, feeling part of the community and availability of health lunches. Pollack Porter et al. [24] observed 1741 children at teenagers across 11 playstreet events. They observed that teenage males were more often observed being physically active than females at the events, and that males were most often seen in areas of the events with sports equipment or facilities. However, within these areas, there were no differences in the amount of physical activity engaged in by males or females. Children were most often active in parts of the event which included inflatables or other general activity areas.

#### 3.4.2. Street Design Interventions

Igel et al. [30] reported a non-controlled pre–post evaluation of decorated footpaths in a deprived district of Leipzig, Germany, which were developed using a participatory approach with local children. Compared to a baseline period, the authors found a greater chance of observing active play on the footpaths. However, the authors reported that no increase in users could be observed. Hence, the footpath intervention was considered as potentially supportive for spontaneous active play ‘on the way’

Biddulph [27] presented their findings on Homezones study in a mainly narrative form using a non-controlled post-test evaluation. They conducted limited observations and found the streets were used by a wide range of community members. They observed 40% of pre-school children and 50% of children are actively playing in homezones, but found that very few teenagers engaged in active play in these zones. They found greater numbers of pre-school children and children spending longer in the spaces compared with adults, highlighting the impact of investment in shared space that is designated as car free. Although the costs of these permanent interventions were not specified, the author emphasises that a low budget investment, rather than expensive surface treatments might be just as impactful. The author’s insight into urban design principles is apparent in his recommendation that such interventions are located in streets that are well connected to well-used routes.

#### 3.4.3. Walk to School Technology Interventions

A pilot non-randomised controlled evaluation of the Beat the Street intervention [28] was inconclusive, finding a small but significant negative effect of the intervention on levels of moderate to vigorous physical activity in school children, with those in the intervention group reporting on average 7 min less than those in the control group. They found some evidence of a significant effect with engagement whereby moderate to vigorous physical activity on days when participants actively swiped a beat box sensor were higher, although these effects were small with a cumulative effect of 3.5 min of activity per day across morning and afternoon commutes for children who engaged in the intervention on an average of 14.5 days. However, as this was a pilot study (N = 80 children) it was not powered to find significant effects and thus results should be interpreted with caution. Hunter et al. [32] did not include a control group in their evaluation but reported data from 3817 children who registered to use the swipe cards. Over a four week period they found that the number of walks registered by the swipe cards decreased from 29% in week 1 to 12% in week 4, which the authors noted could be attributed to the timing (at the start of the school year in the autumn, meaning that there was a short lead-in time for the project), and the lack in some instances of clarity regarding roles and responsibilities. A sub-sample of N = 1025 reported questionnaire data ad baseline and post intervention. The figure reporting walking to school at least once a week rose from 77% to 86% post intervention. Both studies, as would be expected, emphasised the importance of incorporating exercise in a child’s daily routine.

## 4. Discussion

This study aimed to review the impact of interventions modifying the built environment at a street level on children’s health. Despite the increasing recognition of the importance of the built environment for children’s health we found there was limited literature exploring street-level built environment interventions. While recognising the complexity of undertaking studies in the urban built environment, given the challenge of isolating the spatial variable from the myriad other factors that may shape outcomes, we found that many studies were at risk of bias due to study designs lacking a comparator group, or being without baseline measurements. However, of the literature reviewed, it is possible to tentatively conclude that street closure interventions are related to an increase in physical activity or play amongst children. It could additionally be inferred that street closure interventions can have positive impacts in increasing the availability of safe public spaces in deprived settings. There was insufficient evidence to generalise from the results of street design interventions, or interventions that added technology to the local environment to ‘gamify’ active travel to school.

It was evident that although the interventions reviewed aim to improve children’s experience of the street by altering the built environment, there was a lack of description of the specific built environment attributes that relate to the characteristics of the street and to the contextual area where the intervention was to be implemented (e.g., whether it is a residential or a mixed-use street, whether it is a local street or a main road, what type of buildings or land uses are in the block, whether it was shaded or not, and so on). The ten interventions found here highlight this point further: while the studies all captured demographic and socio-economic data in a reasonably consistent manner, the physical setting of the interventions, the specific built environment characteristics of their location, and contextual factors, were rarely described in a consistent way. So, for example, the Beat the Street interventions mention motorised traffic levels, but there is no information on where the children lived in relation to the school, what routes they took, and to what extent fear of traffic impeded their participation. Even maps of the intervention study locales were rarely provided, (the only exception was [27], who provided sketch maps and detailed plans of the designed interventions). Indeed, ref. [31] state that building in GPS and GIS (namely geolocation and spatial analysis) of interventions would “greatly benefit” future research as it would “account for children’s location, enhancing the accuracy of the estimation of the intervention’s contribution” [31] (p. 13). Moreover, beyond the changes to the built environment, street closure interventions were often multi-component, however, other key information such as other activities taking place, level of community engagement, and costs, were often not reported. This lack of detail when describing the intervention in terms not only of what is being done, but also where it is being done and why, poses yet another barrier against replicability. For example, lack of consistency was observed for reporting of the socio-economic characteristics of the area and street where the intervention took place and whether the interventions aimed specifically to address safety or accessibility issues in the area. Consequently, safety and improved access to public spaces or to community resources was reported more as an additional benefit than as an achieved aim. Future research should ensure comprehensive reporting of the built environment context in which intervention studies are located to fully contextualise their effects.

In terms of considerations for upstream planning of the built environment, although the evidence from the studies included in this review is not particularly robust, the studies suggest, as it has been found in previous reviews, that there is scope to widen intervention types. Rather than focusing on permanent changes to physical infrastructure or radical transformations of the built environment for eliciting healthier behaviours, the evidence suggests that soft (namely removable) and temporary measures can deliver increases in positive health outcomes such as play, physical activity and increases in social connections while the intervention is in place. Caveats remain regarding whether there are benefits to the entire population or whether the interventions that are positive for children are also positive for teenagers. Similarly, our review shows that a question remains about whether the studied interventions can have a greater positive impact if the community is actively engaged in the design and delivery of the intervention. Evidence from the public health field has suggested that community led and/or delivered interventions are effective at improving a range of health outcomes [34]. From the studies we reviewed in relation to changing the built environment, it seems plausible that increased community engagement could not only result in even better health outcomes, but also in positive process outcomes related to strengthening the social capital in the community alongside an increased sense of ownership of the interventions. Indeed, research suggests that not only are real-world changes in the built environment important in soliciting more reliable evidence, by involving the communities in which behaviour change is sought, a greater attention to the wider socio-cultural context will be held [35], improving the likelihood of impact [34].

One of the unique aspects of our review relates to our approach to describing the interventions. We used the TIDieR framework [33] as a concise and comprehensive reporting structure. In addition, we expanded the framework in two ways. Firstly, we applied a systematic approach to categorising the content of interventions that effectively expanded the TIDieR framework with our previous categorisation of key built environment indicators to measure in studies of child health [14]. We incorporated the targeted built environment categories in the TIDieR framework as we propose that the description of interventions needs a more precise account of the anticipated changes to the built environment. This description of the built environment changes can be approached in a systematic manner by following the 10 key categories relevant to children’s health [14]. Secondly, we included more detail regarding the level of community engagement for the design and implementation of the intervention as we assessed this was needed in order to capture clear evidence on the study’s setting in order to enhance the replicability of the intervention. Finally, our updated reporting frameworks explicitly describes the level of engagement and results by sex, ethnicity and different age categories (especially differentiating children and teenagers), however, we found these details were rarely reported in the reviewed studies, which highlighted that greater precision in the reporting of these items was needed.

We found a wide range in the quality of evaluations assessed against standard checklists, with few studies that could be classified as ‘high quality’. It is notable that despite concerns with the design of built environment intervention evaluations raised by reviewers in 2015 [17,20], there has apparently been limited advancement in the field. We must acknowledge that evaluating street-based interventions, especially temporary ones, is very challenging. Following the strict rules that are commonly used for public health evaluation where the randomised controlled trial is seen as the ‘gold standard’ [36] is challenging on a number of levels. Built environment interventions can rarely be randomised, and researchers are often dependent on external partners to implement such interventions, making it hard to control research timelines if unexpected factors hinder progress. Selection bias can also be an issue. For example, if the built environment is improved to provide more opportunities for physical activity, this may result in those individuals who are already more active moving to the neighbourhood, making it difficult to ascertain if the intervention has increased activity in those not already active [37]. However, there are opportunities to strengthen the evidence based in this area. We encourage researchers when designing evaluations to consider including control sites where possible, ideally matched by key neighbourhood characteristics such ethnicity, socio-economic status and built-environment characteristics (e.g., walkability); to include both baseline and multiple follow-up data collection to explore whether interventions can effect change over the longer term; to use standardised tools to assess health related outcomes; and to include qualitative approaches to consider the context and mechanisms which might affect the success or failure of interventions [38]. These suggestions will facilitate understanding of the potential longevity of the intervention, and could also serve as a proof of concept to highlight the value or the need associated with making the intervention (e.g., a street closure) permanent, for example by pedestrianizing certain streets.

However, even the best designed evaluations will have limited replicability if the key features of the intervention are not adequately described. We therefore recommend use of the adapted TIDieR framework which incorporates a detailed description of how the built environment is modified, using the 10 indicator list reported in our recent meta-narrative review of the associations between built environment measurements and child’s health (Ortegon-Sanchez et al., 2021). In addition, the extent to which communities are involved in the design (and maintenance) of built environment interventions should be reported, along with costs of delivery. We also acknowledge that in many cases the practitioners implementing the interventions might not have the resources to allocate to conducting monitoring and evaluation, which is why we are suggesting an adapted TIDieR framework as a simple, yet thorough, tool to start the process of presenting comprehensive and systematic descriptions of the key elements of these types of interventions. We invite others to build upon our proposed framework in future research and implementation to aid standardisation of reporting in this field. Where possible, we urge those funding built environment interventions to ensure that there are resources for conducting evaluations using qualitative or quantitative methods (for example, controlled pre and post evaluations using standardised outcome measures).

We mentioned at the start the persistence of built environment obstacles to poor health, and how deprivation is disproportionately aligned with an impoverished built environment. A recent paper highlights our concluding point: that the social determinants of health “are socially distributed and that their influence on health may not be equal across socioeconomic groups” [39] (p. 999)—importantly, the physical characteristics of children’s home environment is more significant than for the population at large. While the authors highlight the quality of housing and the importance of access to a garden, it is clear from our own research that the outdoor surroundings of home are just as important, especially in areas where indoor and private play spaces are limited. Thus, a focus on interventions in such environments would help even out the unevenness of children’s environments and help improve their long-term health outcomes. Policy and decision-makers should work with communities to prioritise built environment interventions in areas of higher deprivation, to provide communities with safe, accessible, well maintained and welcoming environments which promote healthy behaviours. Moreover, policy makers should focus on establishing close collaborations with the communities in deprived areas to, as much as possible, facilitate the co-production of these health promoting interventions so that the communities can shape the interventions to address their needs, and so that they feel a sense of ownership of the intervention which will, most likely, lead to better outcomes.

## 5. Strengths and Limitations

Our study had a number of strengths. It was conducted by a multi-disciplinary team incorporating expertise from the built environment, transport and public health. It focused on interventions at the ‘street’ level in order to capture the most meaningful aspects of everyday use of the children’s local environment and applied a standardised approach to describe the content of interventions in order to aid identification of key intervention ingredients. However, there were some limitations. We found limited published evidence in this area, with all identified studies focusing solely on physical activity or play outcomes. Indeed, many studies are also inconsistent in the way they measure physical activity, for which standardised and validated measurements would be additionally beneficial (Sones et al., 2019). There were also limitations with the design of evaluations and description of interventions which made it difficult to assess their impact. Our review focused on published literature and did not include grey literature where it is possible there could be other examples of built environment interventions. However, given that other authors have highlighted inconsistencies with reporting of interventions and a lack of formal evaluation methods in grey literature in this area (e.g., [40]), we suspect that their exclusion has not impacted on our ability to summarise the state of the literature in this field. We focused our review in high and upper-middle income countries, which means the limited conclusions we can draw may not have relevance to lower income areas.

## 6. Conclusions

Modifying the built environment to improve children’s health offers an exciting opportunity to improve health, especially for those living with deprivation, due to the propensity of impoverished children living in areas which suffer from being polluted, obesogenic, and so on. However, at present there is limited evidence on what types of built environment changes might result in the most significant health improvement. We found the current state of literature to be narrow in focus, with many methodological weaknesses relating to intervention description, evaluation design and the selection of outcome measures. From our review, we can tentatively infer that street closure interventions may be effective in increasing physical activity and play in children. It seems likely also that interventions that involve the local community in the design stages are more likely to affect change. We can also conclude that the state of intervention evidence in this area is sorely lacking, which we suspect is due in part to the difficulties of conducting ‘real world’ evaluations in this area. In the face of competing budget demands, this lack of evidence may limit the confidence of policy-makers in making investments in the built environment to improve health. To overcome these obstacles and build the evidence base in this area it will be important for researchers to work closely with policy and decision-makers at all stages of the planning process. Researchers need to be responsive and flexible in their approach to deal with unanticipated delays or opportunities, and to recognise the time and budgetary constraints of work in policy domains. Policy makers need to commit to involving researchers at an early stage of planning to ensure that before/after testing is made possible so that adequate evaluation of interventions can take place. Together with the focus on evaluation, a focus on systematically reporting the interventions characteristics, costs and identified effects, using a framework as the one suggested in this review, will provide a better understanding of how interventions at the street level can have an effect on children’s health and how they can be replicated. Hence, a commitment to better evaluation and reporting of interventions constitutes an opportunity to shape the pathways to rebalancing the inequalities of children’s health environments.

## Figures and Tables

**Table 1:** Intervention study characteristics.

Study|Origin|Intervention|Quality	Setting	Sample	Outcomes	Design and Analysis
Street closure interventions				
Adhikhari et al., 2021. USA. Play streets (2/10)	One street in a low-income neighbourhood. Columbus, Ohio, USA	N = 69 caregivers of children aged 2–11 (mean age 7) who attended event. 62% of children male N (observed) = 350 children (6 events)	Parent-reported outdoor play (days per week) Parent-reported social connectedness	Cross-sectional, post intervention survey Descriptive analysis
Cortinez-O’Ryan et al., 2017. Chile. Juega en tu Barrio (Play in your neighbourhood) (8/10)	Two neighbourhoods in Santiago. Intervention neighbourhood—85% of population in lower income quintiles. Drug-dealing was common and there had been a recent shooting before the project. Control neighbourhood—93% of population in two lowest income quintiles.	N = 100 Age 4–12 (median age 9 years old for intervention) and 7 years old for control) 51% female 75% low socio-economic position. N (observed) = 293	Objective physical activity (PA): Movband digital pedometer worn over 7 days measuring steps. Parent -reported outdoor play (days per week) Observed physical activity or street use: counts of children in street at key time points during intervention.	Controlled pre-test (pre-intervention)-post-test (last two weeks of the intervention) design Non-parametric inferential statistics (Wilcoxon matched pair, Mann–Whitney U test, McNemar’s test)
D’Haese et al. 2015. Belgium. Play streets (7/11)	19 Play Street projects that lasted 7 consecutive days located within Ghent.	N = 167 children, of which 126 has accelerometer data. Age 6–12 (Mean age 9 years, standard deviation 2 years) 55% male 40% has low family socio-economic status	Objective physical activity -moderate to vigorous (MVPA) Objective sedentary time (ST) (Both measurements assessed via accelerometer worn for 8 days and analysed at intervention times 14.00–19.00 and for the entire day)	Non-equivalent control group pretest (occurring during normal week)-posttest (occurring during playstreet week) design. Design was counterbalanced so ‘control’ condition happened after play street Four level linear regression model was used.
Pollack-Porter et al. 2019. USA. Play streets (3/10)	Chicago, 3rd largest city in US in 2016. Eleven play streets (out of 162 held in summer 2018) included, located in the South region. Target areas were selected for observation.	Age assessed visually by researchers for: child teen, adult or senior. N (observed) = 1741. 1101 children (50% male) and 640 teens (62% male) were observed.	Observed physical activity or street use using SOPARC tool: active or sedentary behaviour.	Descriptive: cross-sectional post intervention; not controlled. Means, standard deviations, and odds ratios reported.
Salazar-Collier et al. 2018. USA. Cyclobias (5/10)	Brownsville, Texas. Town on Texas–Mexico border. One of the poorest cities in the US. Mostly minority city with many low-income residents and documented high rates of disease. 2–3 mile route between parks (4 events)	N (observed) = 5542 participants were observed of which 2577 were children (1646) and teens (931). Age group: child, teen, adult or senior. Adult questionnaire was also distributed (not reported here).	Observed physical activity or street use using SOPARC tool: -by type: cycling, walking, running, other. By intensity: vigorous, moderate, sedentary. (Assessed along route during 15-min intervals within first and third quarter of each hour for which the event was held).	Descriptive; cross-sectional, not controlled. Chi-square test to explore whether physical activity type or intensity varied by age, ethnicity and gender.
Zieff et al. 2016. USA. Play streets (4/10)	San Francisco. Low-income neighbourhoods selected (minimum 16% below poverty line), with higher rates than average of chronic disease, and low levels of recreational amenities. 3 Play Street sites, 1 comparison neighborhood San Francisco, USA	N = 541. 429 children in intervention (38.4%) and 12 in comparison (4.9%). 79 teens in interventions (7.1%) and 21 in comparison (8.6%) Ethnicity (overall sample): Intervention: 23.5% White, 28.1% Black, 30.3% Latino, 18.0% others. Comparison: 11.5% White, 57.2% Black, 16.0% Latino, 12.3% Others	Observed physical activity or street use using SOPARC tool. (Participants’ activities observed for first 15 min of each of the 4 h of play streets). Reported community engagement.	Cross-sectional, controlled observational evaluation with survey. Comparator neighborhoods were selected based on demographic data (race/ethnicity), recreational amenities and health disparities.
**Street design interventions**				
Biddulph 2012. UK. Homezones (4/10)	Seven new-build Homezone schemes with a ‘comprehensive’ range of characteristics.	N (observed) = 420. Pre-school children (64), children (245) and teenagers (111) were observed across the seven schemes	Observed physical activity or street use: ‘Passing through’, ‘active playing’, ‘hanging out’ Time in street: ‘briefly’, ‘a while’, ‘longer’ Social activity: ‘talking’, ‘observing’, ‘not socializing’.	Case study approach. Cross-sectional post intervention; not controlled. Observations of activity/street use studies during 6-h observation period during summer holidays. Numbers of observations reported.
Igel et al. 2020. Germany. Movement enhancing footpaths (7/10)	A large housing estate in Leipzig, with above average unemployment rates, low education levels and below average income levels. Leipzig (Germany)	N (observed) = 929 503 at baseline (114 young children, 276 children and 113 adolescents) 426 observed at follow-up (75 young children, 252 children and 99 adolescents). Young child (0–5 years). Child (6–12 years). Adolescent (13–18 years).	Observed physical activity or street use using SOPARC tool. Categorised into 1: vigorously active and 0: sedentary/walking.	Natural experiment pre-test (baseline), post-test. Each footpath was observed by trained staff on three days (two weekdays and one Sunday) during school term before (T0, August 2019) and after (T1, Sept/Oct 2019) the changes. Multivariate logistic regression analyses reported.
**Walk to school technology interventions**				
Coombes and Jones, 2016. UK. Beat the street (8/11)	Three neighbourhoods in the city of Norwich, covering area approximately 5.7 km^2^ Two primary schools took part. One in intervention area, and one approximately 7.5 km away on other side of the city. The intervention took place across 9 weeks.	N = 80 children aged 8–10 years old Intervention: N = 51 (62.7% female) Control: N = 29 (41.4% female).	Objective physical activity -moderate to vigorous (MVPA) during school days: (Assessed via ActiGraph GT1M accelerometer). Self-reported travel to school: mode to and from school (assessed via travel diary). Engagement measure: number of times each study participant touched a beatbox with smart card.	Pilot non-randomised controlled evaluation Three time points: baseline (week 0), during intervention (week 7), post intervention (week 20) Multiple regression models adjusting for sex, school year, baseline physical activity level, baseline device wear time and change in device wear time between baseline and post intervention. Conducted an ‘intention-t—treat analysis’ and a ‘per-protocol analysis’ which included an engagement measure.
Hunter et al. 2015. UK/Canada. Beat the street international competition (4/11)	Included 12 primary and secondary schools from three cities (London and Reading in UK, and Vancouver in Canada). Walking routes to/from school for 12 primary schools in the three cities.	N = 3817 children aged 9–13 (mean age 11.5 (SD 0.7)). 8% recruited from Vancouver, 66% London, and 26% Reading. N = 2068 provided questionnaire data at baseline and N = 1025 at post intervention. UK Figures only: 55% female, 50% White, 13% Asian, 8% Black, 29% other.	Objective travel to school: Number of walks to and from school assessed via the smart card technology. Self-reported travel to school: mode of travel, attitudes towards walking, active travel and social aspects of physical activity.	Uncontrolled pre- and post- mixed methods evaluation Primary outcome (number walks) assessed continuously through 4-week intervention. Survey measures assessed at baseline, and week 4 (immediate post intervention). Descriptive statistics

**Table 2:** Summary of key street closure interventions characteristics.

Intervention ^a^ Name| ^b^ Aim|^c^ Target Audience	^d^ Street Level Change|^e^ BE Categories	Additional Activities	Frequency/Dose	Who Delivered	Community Engagement in Development	Costs
Adhikhari et al., 2021: ^a^ Play streets ^b^ To create safe opportunities for outdoor play ^c^ Children aged 5–17	^d^ *Temporary:* Closure of residential street block to traffic ^e^ 1,2,3,4	Various: sports, demonstrations, health screening, free healthy meals.	Every two weeks for 3 h over a two-month period. Total of 4 sessions.	Volunteers to staff the events, police to patrol	Unclear: Local stakeholders were engaged before event.	Not reported. Intervention funded by Healthy Neighbourhoods
Cortinez-O’Ryan et al., 2017: ^a^ Juega en tu Barrio’ (Play in your neighbourhood) ^b^ To change the neighbourhood’s social and physical environment, and individual behaviours in order to increase physical activity and opportunities for play ^c^ Families with children living in the area	^d^ *Temporary:* Closure of four residential street blocks to traffic with traffic cones and wardens ^e^ 1,2,3,4	Monitoring of behaviour, play materials (e.g., skipping rope, balls, kites) given to children. Group games organised. Communities provided additional activities.	Twice a week for 12 weeks for 3 h between 17.30–20.30. 26 sessions planned; 24 were delivered.	Local community organisation (CicloRecreoVia) and volunteers from local community to turn away cars.	Intervention tailored to local community preference. Meetings were held with neighbours and stakeholders to obtain input on feasibility, acceptability and design. Strategies proposed were included.	The overall intervention cost (resources, uniforms, stewards and coordinator fees) for the 26 sessions was US $2275.
D’Haese et al. 2015: ^a^ Play streets ^b^ To change the neighbourhood and social environment to provide safe places to play to increase physical activity and reduce sedentary time. ^c^ Families with children living in the area	^d^ *Temporary:* Closing residential street to traffic using fences/signs ^e^ 1,2,3,4	City council offers a box with play equipment that can be hired for free during the intervention period. Box includes balloons, flags chalks, sport equipment. Other equipment also available including trampoline, bouncy castle. There is option to apply for one organised activity.	Dependent on community preference. Street can be playstreet for up to 14 days during summer vacation. Duration between 1400–1900	Local community members. Insurance provided by council	Community led intervention. Volunteers have to make an application to apply. Majority of households in the street have to agree with the application. Communities can also organise their own activities (e.g., barbeque).	Not reported
Pollack Porter et al. 2019: ^a^ Play streets ^b^ To close streets to create safe places and free opportunities for active play. ^c^ Families with children living in the area	^d^ *Temporary:* Closure of street to traffic to facilitate play ^e^ 1,2,3,4	Various activities which varied according to location: for example, DJ for dance area, inflatable play spaces, games. Local services were also present at some offering health screening.	Implemented on one day for 3–5 h and were in summer months. A total of 162 play streets were implemented in 2018.	Planning of play streets was facilitated by two commissioned organisations, funded by the Chicago department of public health. These organisations supported local hosting organisations (local neighbourhood organisations) to apply for play streets in their area, including seed corn funds for organisation and activities. Support in programming activities was also provided.	Intervention was delivered by local hosting organisations. No further details given.	Seed grants of between US $4000–5000 paid to two delegate agencies who then selected hosting partners in their respective regions. From this budget, delegate agencies provided hosting partners with seed grants of up to US $1000 to cover staff stipends, food, and money for materials (e.g., jump ropes). In-kind donations were also received.
Salazar-Collier et al. 2018: ^a^ CycloBia ^b^ To close streets to motorized traffic to allow residents the opportunity to engage in physical activity freely. ^c^ Local Residents	^d^ *Temporary:* Closure of 2–3-mile route to motorised traffic, connecting 4 city parks ^e^ 2,3,4	Physical activity hubs in the city parks offer alternative activities such as free group exercise classes, live music, health concessions and rest areas.	Held between 4–6 times a year on selected streets. Streets closed for 4 h on Friday nights in spring/summer and Sunday afternoons during autumn/winter	The event was hosted by multiple departments and leaders of the city including the mayor, commissioners, Traffic Department, Health Department, Parks and Recreation Department, Police Department, and Transportation Department.	Mentions that the events were supported by a community advisory board, composed of >200 organisations and individuals.	Not reported
Zieff et al. 2016: ^a^ Play streets ^b^ Temporarily closing urban streets to vehicular traffic to provide open space for children and youth to play and increase youth activity time ^c^ Pre-teen youth living in the area, but was open to all	^d^ *Temporary:* Temporary closure of 1–2 street blocks to traffic ^e^ 1,2,3,4	A range of organised activities were provided by the event organisers. Local communities were also encouraged to implement their own activities.	Held at weekend, length of closure not specified. Total of four events held in summer 2013.	Partnership of non-profit organisations in the San Francisco area.	Communities were involved to varying degrees in different communities—in some areas, additional activities were organised, in others, no further activities took place.	Not reported. The Play streets were funded by the Partnership for a Healthier America who selected San Francisco as a pilot site.
**Street Design Interventions**						
Biddulph 2012: ^a^ Homezones ^b^ To redesign streets to prioritise people and not traffic to make them safe places to live and play ^c^ Families living in the area	^d^ *Permanent* Shared surfaces with no clear priorities for cars/pedestrians, natural and non-natural street features/furniture, areas for people to sit, house frontage ^e^ 4,5	None	N/A	Local developers	Not reported	Not reported
Igel et al. 2020: ^a^ Movement enhancing footpaths ^b^ To create attractive places for physical activity (PA) and social interactions and changing social norms with respect to PA and active play in the public sphere. ^c^ Young children who use footpaths	^d^ *Permanent* Decorations (labyrinth, ‘mirror me’, hopscotch grid) implemented on two footpaths. ^e^ 4,5	Not reported	N/A	Implemented by the GRUNAU moves community-based health project.	Followed a participatory planning process with 140 students from two primary schools and a landscape architect. Together they developed and piloted the designs. Children voted on the final selection.	Not reported
**Walk to school Technology Interventions**						
Coombes and Jones 2016: ^a^ Beat the Street ^b^ To ‘gamify’ physical activity and encourage active travel to and from school ^c^ Primary school children.	^d^ *Temporary* Beat box sensors attached to key outdoor locations ^e^ 4	Competition between schools to win prizes. Promotion events. Behaviour change techniques: feedback on performance, setting goals, monitoring progress, encouraging comparison, rewarding positive behaviour.	Daily over a nine-week period	Schools were key delivery partners	Not reported	Not reported
Hunter et al. 2015: ^a^ International walk to school competition (with beat the street) ^b^ To use an international competition to encourage active travel to school ^c^ Primary and secondary school children	^d^ *Temporary* Sensors attached to lampposts at public transport links and school gates marking walking routes around 1 km in length ^e^ 4	International competition based on points accumulated by swiping card against sensors on route to school. Incentive: donations to charity based on points accrued. Prizes donated by local businesses. Participants could get feedback on behaviour via a website.	4 week long intervention	Technology developed by a health IT company. Competition implemented by the project team.	Schools could provide their own in-house rewards. No further detail on community engagement provided	Not reported

Built Environment (BE) categories targeted: 1—Availability or proximity to public open spaces, 2—Safety from traffic and crime, 3—Traffic levels, 4—Social support and psychosocial factors, 5—Pedestrian infrastructure/street environment design.

## Supplementary Materials

The following supporting information can be downloaded at: https://www.mdpi.com/article/10.3390/ijerph19095227/s1, Supplementary Material S1 Full search strategies for all databases. Supplementary Material S2: Table S2.1: Quality Assessment Criteria and Results; Table S2.2: Adapted quality assessment results; Supplementary Material S3: Table S3: TIDieR analysis of the interventions. The Template for Intervention Description and Replication (TIDieR) a standardised checklist and guide commonly used to analyse health interventions; Supplementary Material S4: Table S4: Effect of interventions.
