# Supplementary material for: Shaping Pathways to Child Health: A Systematic Review of Street-Scale Interventions in City Streets
Source: Int J Environ Res Public Health. 2022 Apr 25;19(9):5227. doi: 10.3390/ijerph19095227 (PMC9105466; doi:10.3390/ijerph19095227)
Supplement: Supplementary file 1 [file ijerph-19-05227-s001.zip › Supplementary Material S2.pdf]

Supplementary Material 2:

Table S2.1 Quality Assessment Criteria and Results

Adapted Twohig-Bennett and Jones risk of bias and study quality tool for intervention studies (As reported in Hunter et al)

**Item**<br>**Methodological quality**	**Description**	**Scale**
1. Reporting: hypothesis	Is the hypothesis/aim/objective of the study clearly described?	1: Yes – clearly described<br>0: No
2. Reporting: outcome(s)	Are the main outcomes to be measured clearly described in the introduction or methods section? (if the main outcomes are first mentioned in the results section, this question should be answered no)	1: Yes – clearly described in introduction/methods<br>0: No – not clearly described/first mentioned in results
3. Reporting: intervention	Are the interventions of interest (built environment and control or otherwise) clearly described?	1: Yes – clearly described<br>0: No
4. Randomisation	Was there sufficient description of a randomisation process or statistical test to show that comparability between the two groups has been adjusted for (no explanation scores zero)?	1: Yes – description of a randomisation process<br>0: No – no explanation / no randomisation
5. Exposure	Did the authors show that there was no evidence of a concurrent intervention which could have influenced the results (no explanation scores zero)?	1: Yes<br>0: No – no explanation<br>N: Insufficiently described
6. Representativeness	Were the study samples shown to be representative of the study population?	1: Yes – shown to be representative<br>0: No – shown not to be representative<br>N: Insufficiently described
7. Comparability	Were baseline characteristics of the intervention comparable with the control or were potential confounders at baseline approximately adjusted for in analysis?	1: Yes<br>0: No<br>N: Insufficiently described
8. Attrition	Were numbers of participants at follow-up identifiable as at least 80% of the baseline?	1: Yes<br>0: No<br>N: Insufficiently described
9. Outcome assessment: tools	Were valid and reliable tools used to assess participant outcomes?	1: Yes<br>0: No<br>N: Insufficiently described
10. Follow-up time scale	Was the length of time to follow up assessment appropriate for the intervention?	1: Yes<br>0: No
11. Precision of the results	Were confidence intervals or p-values given?	1: Yes<br>0: No

Table S2.2 Adapted quality assessment results

**Reference**	1. Reporting: aims/hypothesis	2. Reporting: outcome(s)	3. Reporting: intervention	4. Randomisation	5. Exposure	6. Representativeness	7. Comparability	8. Attrition	9. Outcome assessment tools	10. Follow-up time scale	11. Precision of the results	**Total score (out of 11)**
Adihakar et al 2021	1	0	1	0	0	N	0	N/A	0	0	0	2/10
Biddulph et al 2012	1	1	1	0	0	N	0	N/A	0	1	0	4/10
Coombes et al 2016	1	1	1	0	0	N	1	1	1	1	1	8/11
Cortinez-O-Ryan et al 2017	1	1	1	0	0	N	1	1	1	1	1	8/11
Dhaese et al 2015	1	1	1	0	0	N	1	1	1	0	1	7/11
Igel et al 2020	1	1	1	0	0	N	1	N/A	1	0	1	7/10
Hunter et al 2015	1	1	1	0	0	N	0	N/A	1	0	0	4/10
Pollack-Porter et al 2018	0	1	1	0	0	N	0	N/A	1	0	0	3/10
Salazer et al 2018	1	1	1	0	0	N	0	N/A	1	0	1	5/10
Zieff et al 2016	1	1	1	0	0	N	0	0	1	0	0	4/10
