# Supplementary material for: Shaping Pathways to Child Health: A Systematic Review of Street-Scale Interventions in City Streets
Source: Int J Environ Res Public Health. 2022 Apr 25;19(9):5227. doi: 10.3390/ijerph19095227 (PMC9105466; doi:10.3390/ijerph19095227)
Supplement: Supplementary file 1 [file ijerph-19-05227-s001.zip › Supplementary Material S3.pdf]

Supplementary Material S3:

Table S3. TIDieR analysis of the interventions. The Template for Intervention Description and Replication (TIDieR) a standardised checklist and guide commonly used to analyse health interventions (Cotterill et al., 2018)

**Paper title**	**An evaluation of PlayStreets in the South Side neighbourhood of Columbus, Ohio (Adhikhari et al 2021)**
**1. Brief name**	**Playstreets**
**2. Why**	Outdoor play important for children's health as it can increase levels of physical activity. Safety concerns can be an issue for families living in disadvantaged neighbourhoods and means that families are less likely to spend time outdoors. PlayStreets aim to increase children's physical activity, instill a community culture of outdoor plan and improve social interactions in communities. Previous studies have suggested these types of interventions can increase physical activity on the days they are implemented. They are typically hosted by non-profit or civic organisations and involve closing off a neighbourhood street to traffic and hosting events and activities aimed at children. Aim of intervention was to improve neighbourhood perceptions and create safe opportunities for outdoor play. The current intervention was targeted at children aged 5-17.
**3. What – material**	Play equipment to be used when PlayStreets were active and leaflets to promote the Playstreet.
**4. What – procedures**	The Playstreets featured a range of activities including sports, toy cars/ scooters and arts and crafts. There was also a unique featured activity each week, these included a hip-hop dance class, story time, bike demonstration, a bounce house, and a petting zoo. Free health screenings and health information were offered via a mobile health unit at two events.<br>Free healthy meals were provided to all attendees and volunteers.
**5. Who provided**	Funding for the events was secured by the Healthy Neighbourhoods Healthy Families initiative, led by the National Children's hospital and an academic research centre based within a hospital. The PlayStreets were staffed with volunteers from NCH and neighbouring high schools.<br>The local police division provided security, guarded the streets to prevent cars obstructing the play area and patrolled regularly.
**6. How**	A block party permit was obtained. Local stakeholders (e.g. community development organisation, public library, public health department and two civic associations) were engaged before the event and agreed to share information with residents. Playstreets were advertised using targeted marketing via facebook, mail-outs to families with children living in the area, door-to-door outreach and distribution of flyers via local businesses, playgrounds and not for profit organisations.
**7. Where**	One block within the South Side neighbourhood in Columbus, Ohio
**8. When and how much**	Intervention was implemented in the Summer of 2019 between mid-June and mid August. PlayStreet was implemented every other week and involved closing the street between 10am-1pm
**9. Tailoring**	N/A
**10. Modifications**	N/A
**11. How well – planned**	N/A
**12. How well – actual**	N/A

**Paper title**	**Radical streets? the impact of innovative street designs on liveability and activity in residential areas** (Biddulph, 2012)
**1. Brief name**	**Homezones**
**2. Why**	Based on concept of liveable streets which explores how streets can influence the behaviours of individuals encouraging healthy behaviours (e.g. physical activity outdoors) and discouraging

	unhealthy or unsafe behaviours (e.g. speeding traffic). It bases assessment of streets using the Woonerf Design criteria (first developed in the Netherlands) which views streets as social spaces and prioritise shared spaces and low traffic speeds. The aim is to redesign streets to prioritise people and not traffic to make them safe places to live and play.
**3. What – material**	Natural and non-natural street features / furniture are aimed at traffic calming within immediate area
**4. What – procedures**	. Physical changes to the environment: housing developments where streets have 'shared surfaces' so that there is no clear priority for cars or pedestrians; generally houses front doors face towards street creating a community feel; natural (e.g. trees, planters) and non-natural (e.g. bollards, stone features) features/furniture in street; informal (e.g. low walls) or formal (e.g. benches) areas for people to sit.
**5. Who provided**	Developers in conjunction with local authority who approve plans.
**6. How**	N/A. This was assessment of interventions in place.
**7. Where**	New urban housing developments across the UK
**8. When and how much**	No further input once development is built.
**9. Tailoring**	None
**10. Modifications**	N/A
**11. How well – planned**	N/A
**12. How well – actual**	N/A

Paper title	Gamification of active travel to school: A pilot evaluation of the Beat the Street physical activity intervention (Coombes and Jones, 2016)
1. Brief name	Beat the Street
**2. Why**	Building on the popularity of gaming amongst young people this interventions seeks to 'gamify' physical activity. This approach seeks to transfer the 'addictive' elements of games to health behaviours to promote formation of new healthy habits (Cugelman, 2013). This is done by building in game like elements to the behaviour such as receiving rewards, and encouraging competition. Key behaviour change techniques include: providing feedback on performance, setting goals, monitoring progress, encouraging comparison of behaviour with others, and rewarding positive behaviour.
**3. What – material**	Equipment: 'Beat boxes': sensors attached to key outdoor locations (e.g. lamp-posts) in local neighbourhoods; smartcards that are distributed to participants allowing them to 'clock-in' at the sensor, participants earn a point for each time they clock in with a sensor. 40 beat boxes were deployed across three neighbourhoods (5.7km2) in locations that were easier to reach via active travel means than via cars. Other materials including prizes for competitions.
**4. What – procedures**	Participants were awarded a point each time they touched their smartcard on a sensor, allowing children to compete against other pupils at their school to see who could achieve the most points. Distances between sensors were computed to provide estimates of distances walked and cycled and targets were set to 'walk and cycle around the world'. Schools competed against each other with a prize for a school with the most points after 9 weeks competitions. Prizes included funding to spend on sports equipment, books or resources. Schools were also able to compete against other groups taking part in Beat the Street, such as local workplaces, for weekly spot prizes donated by local businesses. Promotion events took place regularly to promote interest and encourage participation.
**5. Who provided**	Unclear who commissioned the intervention. Schools were key delivery partners in the intervention. No details of training given.
**6. How**	Smart cards provided individually to participants, but were also encouraged to act as teams within schools. Unclear from report how participants were instructed in how to participate in the intervention.

**7. Where**	Local neighbourhoods with primary school as a focus. Beat Boxes were installed on lamp-posts across the neighbourhoods.
**8. When and how much**	Intervention was delivered over a nine week period. Participants were encouraged to clock with with beat boxes on a daily basis.
**9. Tailoring**	N/A
**10. Modifications**	N/A
**11. How well – planned**	The intervention made use of regular promotion activities to increase engagement and participation; these included competitions between participating schools, and other work-places for prizes donated by local businesses.
**12. How well – actual**	84% of children in the intervention group took part in the intervention (e.g. touching a beat box at least once). Over 9 weeks, these participants touched a beat box on an average of 15 days, and made a total of 78 swipes per child (average 5 swipes per day).

**Paper title**	**Reclaiming streets for outdoor play: A process and impact evaluation of "Juega en tu Barrio" (Play in your Neighbourhood), an intervention to increase physical activity and opportunities for play** (Cortinez-O'Ryan et al., 2017)
**1. Brief name**	**Play in your Neighbourhood**
**2. Why**	Unstructured play in children is thought to promote greater levels of physical activity as well as healthy learning, mental health and growth. However, social and physical environments can constrain opportunities for children to engage in play and spontaneous activities. Improving physical environments to make them more conducive to play may increase levels of physical activity amongst children. [INCLUDE AIM HERE]
**3. What – material**	Individual materials: Information leaflet and magnetic calendar used as a monitoring device for recording activities, play materials (e.g. skipping rope, balls, kites) were given to each child. Community 'traffic enforcers' (who were there to turn away cars from the streets) had identifiable uniforms and signs for traffic. Physical barriers and cones were used to block off traffic from streets. The overall intervention cost (resources, uniforms, stewards and coordinator fees) for the 26 sessions was US$2275.
**4. What – procedures**	Four continuous blocks were closed to motorized traffic from 17:30 to 20:30 each Wednesday and Friday. The temporary road closures had local authority approval. A website, logo, and Facebook page were created to promote the intervention. At an individual level participants were encouraged to engage in activities and record these use their magnetic diary. Local monitors led group games and incentivized children to meet each other during the first four sessions. Some neighbours took on this role while others provided music and organized contests in some sessions. The social and physical environment was modified through street closures organized by experienced stewards from CicloRecreoVia, who were located at each intersection. Wearing identifiable uniform and using special traffic signs they rerouted traffic, kept the street free from parked cars, and alerted other adults if any problems arose. They placed physical barriers and cones at intersections.
**5. Who provided**	Intervention was delivered by a local community organisation (CicloRecreoVia) who were experienced in these initiatives. Local communities also helped to implement the intervention.
**6. How**	Face to face intervention, delivered to individuals and groups. Information was also available via a project website.

**7. Where**	The intervention neighbourhood was located in a mixed land use-area in Santiago, Chile, with 17,347 inhabitants/km2, of which 24% were children. Eighty-five percent of its population were in the two lowest income quintiles. A gated community with six, four-story apartment buildings was located at one end of the neighbourhood. Drug dealing activity was common in the street and a shooting occurred nearby before the intervention began. JETB begun.
**8. When and how much**	The intervention was implemented twice a week for 12 weeks between 17.30-20.30 on Wednesdays and Fridays.
**9. Tailoring**	The intervention was tailored to local community preferences. Local communities were involved in developing the intervention and strategies that they identified were included. These included activities such as street cleaning (stones, pieces of glass, street dogs), posting advertising posters, and providing activation aids such as music, facilitators, and playing materials.
**10. Modifications**	None
**11. How well – planned**	See below
**12. How well – actual**	Twenty four (92%) of the sessions were implemented as planned. Two diverged from the established design. During the second session only two blocks were closed due to a wake, and during the final session a closure event was held instead of regular closure in which neighbours and district authorities gathered to watch artistic performances. 53% of children in intervention group participated 'often or always' in sessions (more than 70% of sessions), 31% participated 'seldom or never' (less than 40% of sessions).

**Paper title**	**Organizing "Play Streets" during school vacations can increase physical activity and decrease sedentary time in children** (D'Haese et al., 2015).
**1. Brief name**	**Playstreets**
**2. Why**	Active outdoor play can contribute significantly to the amount of moderate - vigorous activity children engage in. However various concerns about safety may limited opportunities for outdoor play. Environmental interventions which provide opportunities for outdoor play may be more effective at increasing physical activity than interventions based on individual determinants of behaviour. Since 1998, Play Streets have been organized in different Belgian cities and villages during school vacations and are a collaboration between the inhabitants of the Play Street and the city council. A Play Street is a street that is reserved for children's safe play for a specific period during school vacations. Generally motorised vehicles are banned from the street (and local traffic proceeds at a 'foot' pace) meaning children can play unhindered and safely. Play streets aim to change the neighbourhood environment (by banning vehicles) and social environment (by encouraging social interaction amongst children playing in the street).
**3. What – material**	Materials are provided by the city council. They include fences and traffic signs to block off streets to traffic, and play materials to borrow free for either the duration of the play street (play equipment, for example, balloons, water balloons, flags, chalks, rackets, balls) or for one day (popular games, a trampoline, a bouncy castle, a circus box). These need to be returned to the city council after the intervention finishes.

**4. What – procedures**	Any street inhabitant can apply to the local council for the street to become a play street for a maximum of 14 days during July and August (during school summer vacations). The application is for one period of time only (but can be renewed yearly). After the city council approves the application, the majority of inhabitants in the street need to agree with the approved application. A least three volunteers in the street need to sign an agreement with the council for organisation of the play street – they are responsible for informing the street inhabitants about the timing and rules of the play street. Each day the Play Street is organized, the volunteers enclose the Play Street with fences and a traffic sign, indicating that car traffic is forbidden in the streets. The Play Street can be cancelled by the volunteers on that day. Parents remain responsible for their children playing in the street. The city council regulates insurance for the volunteers. The play street is organised between 1400h-1900h. In additional to free play materials volunteers are free to organise their own events (e.g. barbeque). They volunteers can also ask for one organised activity to be provided by the local council, for example, a circus school.
**5. Who provided**	Intervention delivered by local volunteers who manage the application process and organisation. No details of training given.
**6. How**	Face to face intervention.
**7. Where**	To become eligible to be a play street the following criteria must be met: the street is a residential street, with a speed limit of maximum 50 km/h, there is no significant passing traffic (e.g. public transport), and the surrounding streets remain accessible after the introduction of the Play Street.
**8. When and how much**	The intervention group consisted of 19 play streets that operated for at least 7 consecutive days.
**9. Tailoring**	The intervention is tailored to local community preferences
**10. Modifications**	Not reported
**11. How well – planned**	Not reported.
**12. How well – actual**	Within the intervention group 81% played in the play street during the course of the intervention; 70% reported having access to the borrowed play equipment, but only 25% of parents reported that this equipment was valuable.

**Paper title**	International inter-school competition to encourage children to walk to school: a mixed methods feasibility study. (Hunter et al 2015)
**1. Brief name**	Beat the Street
**2. Why**	Physical activity is important for health, but often declines in adolescence. Increasing physical activity at this time could contribute to healthy habits over a life time. Active travel (e.g. walking or cycling) to school can be an important contributor to overall activity levels, but this type of travel is declining. Novel approaches to encouraging walking and cycling to school are needed. Competition might be an important motivator. Current intervention targeted primary and secondary school children and aimed to see whether an international walk to school competition could increase active travel.<br>Intervention based on Learning Theory and Social cognitive theory.
**3. What – material**	Sensors and swipe cards, incentives and a website.
**4. What – procedures**	International walk to school competition: children could win points for themselves, their school and their country over a 4-week period. Trips to and from school were eligible. Two points were awarded for trips up to 0.5km and 3 points for trips over 0.5km. Children who lived 1-2km from

	school were encourage to walk the whole trip. Those who lived further away were encouraged to get off the bus a stop earlier or stop the car a few streets away.<br><br>Walks were recorded using swipe card technology. Sensors were placed at key points on walking routes to and from school. When children swiped their key card over the sensor this created a timestamp logging the date and time of walk. A bout of walking was recorded when the participant swiped two sensors (e.g. en route, and at the school gate).<br><br>For each point earned by participants, one pence was donated to the charity of the schools choice. 12 prizes were randomly allocated over the competition period, these included a £10 voucher for a local retailer (three vouchers per week) and a family trip to a theme park.<br><br>Participants could get feedback on walking behaviour and their performance in relation to other via a league table on the website. The website also offered other functions including maps of sensor locations, walking routes, and online support forums.
**5. Who provided**	Technology was developed by Intelligent Health Ltd – a health IT company who develop and implement physical activity programmes. It was unclear who liaised directly with schools about implementation of the intervention
**6. How**	Swipecards were given individually to children. Unclear how other elements of the intervention were delivered; some group
**7. Where**	Based in communities around schools set in major cities in England and Canada
**8. When and how much**	4 week long intervention conducted in September / October time (year not specified.
**9. Tailoring**	Sensors could be located in areas identified by schools.
**10. Modifications**	Schools could offer additional prizes if they wished, for example certificates to their top 10 performers.
**11. How well – planned**	Not reported
**12. How well – actual**	100% of those registered to take part used their card at least once during the competition period. 16% swiped card every day, and 35% used their card on 5 or fewer days. 29% who registered walked to and from school every day in week 1, this declined over the four week period.

**Paper title**	"Movement-enhancing footpaths" – A natural experiment on street design and physical activity in children in a deprived district of Leipzig, Germany.(Igel et al 2020)
**1. Brief name**	Movement enhancing footpaths
**2. Why**	Physical activity is important for children's health. Active play is particularly important for young children as it improve physical, emotional, social and cognitive health and contributes to physical activity levels. 'Playable' environments can encourage spontaneous physical activity and social interactions could be important, but there is little evidence about the effect of built environment features on physical activity in youth. The current study was part of a participatory planning project aimed at increasing the physical activity of children. It aimed to do this by creating attractive places for physical activity or active play, and social interactions and changing social norms in relation to outdoor play.
**3. What – material**	Footpath signage
**4. What – procedures**	Planning process which involved 140 students from two primary schools and a landscape architect. Over course of seven lessons children mapped daily route, explored barriers to locomotion and developed ideas about street games to promote vigorous activity. These ideas were piloted in the school playgrounds. They were subsequently were developed into solitary or interactive active play designs by a landscape architect. After negotiations were municipal departments, three decorations (labyrinth, 'mirror me' and hopscotch grids) were implemented

	on two footpaths. One was near a primary school, gym and playground, the other was near a community centre and health centre.
**5. Who provided**	A community-based health promotion project call GRÜNAU Moves
**6. How**	N/A
**7. Where**	Deprived district of Leipzig, Germany
**8. When and how much**	The participatory process started in 2016 and the footpath changes were made in 2019
**9. Tailoring**	N/A
**10. Modifications**	N/A
**11. How well – planned**	N/A
**12. How well – actual**	N/A

**Paper title**	**Transforming city streets to promote physical activity and health equity.** (Pollack Porter et al., 2019)
**1. Brief name**	**Chicago Playstreets**
**2. Why**	People who live in urban areas can face barriers to being physically active because of because of low levels of walkability, lack of access to safe parks and playgrounds, and concerns related to traffic and crime. Creating or improving public places for physical activity is one recommended evidence supported strategy for increasing physical activity; but this is not always easy because of barriers associated with making infrastructure changes such as cost and concerns about the displacement of residents and businesses. In contrast, interventions that temporarily change the physical environment to promote physical activity for a defined time can be a feasible, low-cost strategy to support opportunities for physical activity and active play. Play streets are one such type of intervention which can promote physical activity by providing safe spaces for children to play. They are also though to addresses inequities in access to opportunities for physical activity among youth because they are free to attend and provides safe places for play, especially in communities that lack opportunities for children to be physically active.
**3. What – material**	Mentions materials that are provided by small grants to aid implementation of playstreets including play equipment and activities (e.g. slides, bouncy castles). Marketing materials (e.g. fliers) and expertise in programming events was also available.
**4. What – procedures**	Playstreets initiative was funded by the Chicago department of public health who commissioned two community organisations in the city to provide mentoring and technical assistance for implementation of playstreets, including administration of grants to local organisers of between $4000-5000. Hosting partners in each neighbourhood were selected via a competitive process, these were local organisations (for example, churches, neighbourhood associations). Playstreets were generally implemented on one day (weekend or weekday) for a duration of between 3-5 hours, and were in the summer months. They aimed to provide a localised place to play, free of traffic. PlayStreets offered various activities dependent on their location, including sports clinics or movement instruction (such as Zumba); a DJ for a dance area; inflatable play spaces, including slides and bounce houses; and various games (for example, playing with Hula Hoops or Frisbees). PlayStreets also provided critical access to community and city resources for residents, depending on hosting partners’

	connections to local businesses and organizations. Neighbourhood residents, businesses, community organizations, and other entities donated resources such as giveaways (including school supplies for children), food and drink for attendees, and information that was shared by representatives from local schools and other community organizations (for example, local banks) (exhibit 2). For all PlayStreets that did not receive donations of food or drinks, hosting partners used their supply allowance to ensure that there was adequate food and drink for all attendees. The health, hygiene, and personal care services provided included exams, haircuts, and information about substance abuse or violence prevention. Financial advice by a county treasurer and free legal advice by a representative of the local state attorney's office were provided at one PlayStreet. At another, attendees received access to free health screenings and services, which, according to one hosting partner, included kidney, glucose, cholesterol, and vision screenings; dental care; and free vaccines.
**5. Who provided**	Planning of playstreets was facilitated by two commissioned organisations, funded by the Chicago department of public health. These organisations supported local hosting organisations (local neighbourhood organisations) to apply for playstreets in their area, including seed corn funds for organisation and activities. Support in programming activities was also provided.
**6. How**	Face to face
**7. Where**	Local streets
**8. When and how much**	27 hosting partners implemented 162 playstreets in Chicago in 2018.
**9. Tailoring**	The intervention is tailored to local community preferences as they are organised by local 'hosting' organisations.
**10. Modifications**	N/A
**11. How well – planned**	Not reported
**12. How well – actual**	N/A

**Paper title**	**Evaluation of Event Physical Activity Engagement at an Open Streets Initiative Within a Texas-Mexico Border Town** (Salazar-Collier et al., 2018)
**1. Brief name**	**Cyclobias**
**2. Why**	Open streets initiatives, such as Ciclovías, are events where city streets are temporarily closed to motorized traffic allowing city residents the opportunity to engage in physical activity (PA) freely. They are currently mainly found in Latin America. They are thought to encourage physical activity, which in turn improves health, leading to cost-savings from having to treat less chronic illness in populations. A review of 4 Ciclovias programmes found that they are cost-beneficial, with an event was found to be cost beneficial with a cost-benefit ratio of 2.32 estimated for San Francisco's event, indicating a savings of $2.30 for every dollar invested in the program.
**3. What – material**	An average number of 100 rental bikes are made available free of charge at each event to encourage cycling for all attendees.
**4. What – procedures**	Brownsville, TX's "CycloBias" are held between 4 and 6 times per year on selected streets in the downtown area. The streets are closed for 4 hours on Friday nights during the spring and summer months and Sunday afternoons during the fall and winter months. The 2- to 3-mile route connects 4 city parks, which serve as reclovías or PA hubs, which are open spaces offering alternative activities, such as free group exercise classes, live music, healthy concessions, and rest areas. Prior to each CycloBia, the

	event is promoted through local print, radio, and social media venues, and a press conference is held. Because a coalition approach is taken when implementing the CycloBia event, the various organizations promote the event to their constituency. In addition, flyers are distributed to the homes and businesses near the CycloBia route.
**5. Who provided**	The event is hosted by multiple departments and leaders of the city including the mayor, commissioners, Traffic Department, Health Department, Parks and Recreation Department, Police Department, and Transportation Department. Many other community partners support the event, most of which are part of a Community Advisory Board, composed of over 200 organizations and individuals across the region, focused on increasing PA and healthy food choices.
**6. How**	Face to face
**7. Where**	Streets linking major parks in the city.
**8. When and how much**	4 different CycloBia events held in June, August, November, and December of 2015.
**9. Tailoring**	Not reported
**10. Modifications**	N/A
**11. How well – planned**	Not reported
**12. How well – actual**	N/A

**Paper title**	**Creating neighbourhood recreational space for youth and children in the urban environment: Play(ing in the) Streets in San Francisco.** (Zieff et al., 2016)
**1. Brief name**	**Playstreets**
**2. Why**	In urban areas particularly, space limitations, inadequate financial resources and low prioritizing of health-benefiting recreation, have diminished the opportunities for increasing physical activity among the broader population. Physical activity in children has been associated with built environment features (e.g. access to and use of parks), poorer access to parks is associated with less physical activity. Social environments such as organised activities and connecting with friends, can also encourage activity. Where access to parks or other spaces to play is limited, Joint-use agreements are one effective strategy increasingly being used by communities to support PA by encouraging shared facility use between schools and local organizations or by opening school resources to the local community. Open Streets initiatives, in which miles of streets are closed to vehicle traffic, are another promising mechanism for increasing PA and offering broad range community health benefits. Play streets are an evolution of the open street concept in the US, with the goal of temporarily closing urban streets to vehicular traffic to provide open space for children and youth to play. Play Streets was to be implemented on a smaller-scale (1–2 car-free city blocks) with lower staff requirements and was designed to provide flexibility for each neighbourhood in determining their unique needs, cultural preferences and utilization of resources. The simplified structure of Play Streets with its possibility of more frequent events adds significantly to the amount of open space available for recreational and social activities for the city's youth and children.
**3. What – material**	Play equipment and materials (e.g. climbing wall, bike tracks)
**4. What – procedures**	The San Francisco Recreation and Parks department offered organised activities to the play street, and local communities were also encouraged to implement unofficial organised activities. San Francisco Recreation and Parks Department provided climbing wall and bicycle ramps at each site. The spontaneous activities differed by site because

	of community decision-making around offered programs and included magic show, basketball, soccer, tag, bean bag throw, sidewalk chalk drawing, Zumba, and hula hoops. In addition to providing an important recreational opportunity, Play Streets offered neighbourhood residents an opportunity for social interaction and community building. The communities identified for Play Streets implementation participated in the development, programming, and implementation process in varying degrees. On average, 150 residents attended "town hall meetings" scheduled by the staff of Sunday Streets San Francisco, and voiced both support and concerns about the process, which is an indication of engagement with the project. Community response to the invitation to self-advocate ranged from organization of an additional event by the school parent-teacher association in one neighbourhood a month after the initial event, to the relative absence of community involvement in another neighbourhood.
**5. Who provided**	Play Streets in San Francisco was organized and implemented through a partnership between non-profit organizations (i.e. Liveable City, the umbrella organization of Sunday Streets and San Francisco Beautiful, a local advocacy organization) and the fiscal sponsorship of the San Francisco Municipal Transportation Agency (SFMTA).Play streets were funded by the Partnership for a Healthier America (PHA), who selected San Francisco as a pilot site.
**6. How**	Face to face
**7. Where**	Criteria for neighbourhoods being selected as pilot site for play streets included: low-income (e.g., minimum 16% below poverty line); higher rates than the city average of chronic diseases including childhood obesity; and areas low-served for recreational resources (e.g., less than one acre of open space per 1000 residents, toxic land). In addition, the following criteria were used in relation to selecting streets: excluded streets with public transportation to minimize disruption to service; gave preference to residential streets; and considered features such as steep slopes and availability of facilities. Connection with other neighbourhood resources (e.g., community centres) was also a determining factor.
**8. When and how much**	Four events held in Summer 2013
**9. Tailoring**	The intervention was tailored to neighbourhoods and local communities.
**10. Modifications**	
**11. How well – planned**	
**12. How well – actual**	In all cases, Play Streets added significantly to open space usable for recreation in close proximity to the resident participants (increases between 47-100% in three neighbourhoods).
