# Supplementary material for: Shaping Pathways to Child Health: A Systematic Review of Street-Scale Interventions in City Streets
Source: Int J Environ Res Public Health. 2022 Apr 25;19(9):5227. doi: 10.3390/ijerph19095227 (PMC9105466; doi:10.3390/ijerph19095227)
Supplement: Supplementary file 1 [file ijerph-19-05227-s001.zip › Supplementary Material S4.pdf]

Supplementary Material S4

Table S4. Effect of interventions

Study | Origin | intervention name	Key findings
**Street Closure Interventions**	
Adhikhari et al, 2021 [25]<br>USA<br>**Playstreets**	**reported play:** 55% of caregivers reported that on average their children play outside on 5-7 days a week, and 16% say they play outside 1-2 days a week. If not for the Play Street, 55% of caregivers said their children would be inside, and 45% said they would be playing outside.<br>**reported social connectedness:** 80% of children met new friends at PlayStreets and 54% of caregivers said to have more contact with neighbours.<br>**perceptions of safety:** 92% of caregivers felt that Playstreets made the neighbourhood feel safer.
Cortinez-O'Ryan et al, 2017 [30]<br>Chile<br>**Juega en tu Barrio (Play in your neighbourhood**)	**↑ objective PA:** Significant increase in number of steps on weekdays and during intervention hours, and proportion of children who met recommended PA for intervention group but not control.<br>**↑ parent-reported play:** Significant increase in daily and weekly outdoor play.<br>**↑ observed street use:** Increase in children counted in block from 0 (before) to max 60 (range 29-126) during the 25 sessions (intervention only).<br>**↑ perceptions of safety:** Perception that neighbourhood safe for children to play in daytime increased from 35% to 54%
D'Haese et al 2015 [28]<br><br>Belgium<br><br>**Playstreets**	*Activity during intervention hours (14.00-19.00):*<br>**↑ MVPA and ↓ ST in intervention group:** MVPA was higher (36 vs 27 mins/day) and ST was lower (138 vs 146 mins/day) in intervention week.<br>**↓ MVPA and ↑ ST in control group:** MVPA was lower (24 vs 27 mins/day) and ST was higher (164 vs 156 mins/day) in intervention week vs non-intervention week.<br>*Entire days activity:*<br>**↑ MVPA and ↓ ST in intervention group:** MVPA was higher (67 vs 55 mins/day) and ST was lower (337 vs 367 mins/day) in intervention week.<br>**↓ MVPA and ↑ ST in control group:** MVPA was lower (53 vs 57 mins/day) and ST was higher (400 vs. 381 mins/day) in intervention week vs non-intervention week.
Pollack Porter et al 2019 [22]<br><br>USA<br><br>**Playstreets**	*During the event:*<br>**↑ observed PA of male children and teens (compared to females):** Compared to females, male children (OR:1.38; 95% confidence interval: 1.09, 1.74) and male teens (OR: 1.96; 95% CI: 1.42, 2.71) were more likely to be observed to be physically active..<br>*In target areas classified as sport specific (e.g. areas with basketball hoops):*<br>**↑ observed PA of male teens (compared to male teens observed in all other target areas):** Male teens were more likely to be observed as physically active, compared to male teens in all areas (OR: 2.13; 95% CI: 1.42, 3.20) (data not shown). While a higher proportion of male teens relative to female teens was in the sport-related areas, once there, both sexes were equally physically active (66 percent).<br>*Inflatables and general activity target areas:*<br>**↑ observed PA in children (compared to children observed in all other target areas):** Children, regardless of sex, were more likely to be observed as physically active (74 % and 55 %, respectively), compared to children observed in all other areas (OR: 2.55; 95% CI: 1.98, 3.29).
Salazar-Collier et al 2018 [23]<br>USA<br>Cyclobias	**Type of physical activity varied by age group:** Bicycling: 32.7% were children, 17.9% teens, 48.7% Adults, 0.7% Seniors. Running: 19.7% were children, 25% teens, 55.3% adults. Walking: 20.7% children, 15.6% teens, 62.2% adults, 1.5% seniors.<br><br>**Intensity of physical activity varied by age group:** Vigorous activity: 61.8% were children, 13.6% teens, 24.3% adults, 0.4% seniors. Moderate: 38.6% children, 13.3% teens, 46.9% adults, 1.3% seniors. Sedentary: 25.8% children, 13.4% teens, 57.1% adults and 3.8% seniors.
Zieff et al 2016 [24]	**↑ number of children <14 on streets during Play Streets**<br>The percentage of children below 14 years old on the streets increased from 4.9% on the comparison day to 38.4% during Play Streets.

**Study | Origin | intervention name**	**Key findings**
USA<br>Playstreets	Youth and child participants were predominantly male (62.7%).<br>During 'treatment' weeks, in each play street site there was a greater percentage of young people observed in vigorous activity.

**Study | Origin | intervention name**	**Key findings**
**Street Design Interventions**	
Biddulph 2012 [27]<br><br>UK<br><br>Homezones	**Pre-school children and children observed actively playing and spend 'a while' or 'longer' in Homezones**<br>40% of pre-school children and 50% of children are actively playing in Homezones. Teenagers spend very little time in active play (~2% observed).<br>80% of pre-school and 85% of children spend 'a while' or 'longer'. This figure is greater than number of adults (~45%).<br>**Other findings**<br>Mapping where children played showed that children could roam the streets with relatively freedom and play especially in spaces with low car volumes, low speeds, or car-free spaces connected to the street network
Igel et al, 2020 [29]<br>Germany<br>Movement enhancing footpaths	**↑ chance for vigorous PA foro young children and children**<br>Logistic regression showed a greater change for vigorous activity on decorated footpaths (OR 2.45, CI1.54-3.89) for children aged 0-12 (OR 19.32, CI13.07-28.56) and on Sundays (OR 2.54, CI1.59-4.06)<br>25.6% of the observed young children and 18.6% of the children aged 6 -12 used the colored markings for PA. There were no differences between male and female users<br>**None of the variables was related to PA in adolescents and no increase in users could be observed**
**Walk to school Technology Interventions**	
Coombes and Jones, 2016 [26]<br><br>UK<br><br>Beat the street	*Intention-to-treat analysis (N=80):*<br>**↓ MVPA (weekday evening) in intervention compared to control**<br>The only statistically significant intervention effect was a decline in weekday evening MVPA of 6.5 minutes amongst intervention compared to control school children.<br>*Per-protocol analysis:*<br>**↓ MVPA (weekday evening) in intervention compared to control**<br>The only statistically significant intervention effect was a decline of 7 minutes in weekday evening MVPA amongst intervention compared to control children.<br>**↑ MVPA during school commute linked to engagement**<br>Found an increase in MVPA during school commute associated with number of days children touched the sensor. For morning commute each day a child touched a Beat Box corresponded to additional increase of 8.3 seco and for each afternoon commute an additional 6 seconds<br>**↑ active travel to school in intervention school postintervention**<br>Active travel increased 10% at intervention school post intervention while it decreased 7% at the control school (p=0.056).
Hunter et al (2015) [31]<br><br>UK/Canada<br><br>Beat the street international competition	**↑ objective travel to school in week 1 but ↓ in weeks 2-4**<br>29% of children walked to/ from school in week 1 but a decline was observed (week 2: 18%; week 3: 14%; week 4: 12%).<br>**↑ self-reported travel to school post intervention**<br>At baseline 77% (N=601) walked to school at least once a week compared with 86% (N=672) post intervention.<br>At baseline 68% (N=531) walked on five or more journeys to and from school compared with 76% (N=594) at follow-up.<br>***Other findings***<br>Post intervention, children felt that walking to school helped them stay healthy (97%), feel happy (81%) and stay alert in class (76%)
